# The Impact of Robotics in Learning Roux-en-Y Gastric Bypass: a Retrospective Analysis of 214 Laparoscopic and Robotic Procedures: Robotic Vs. Laparoscopic RYGB

**DOI:** 10.1007/s11695-020-04508-1

**Published:** 2020-03-02

**Authors:** Jan Henrik Beckmann, Alexander Bernsmeier, Jan-Niclas Kersebaum, Anne-Sophie Mehdorn, Witigo von Schönfels, Terbish Taivankhuu, Matthias Laudes, Clemens Schafmayer, Jan-Hendrik Egberts, Thomas Becker

**Affiliations:** 1 Department of General, Visceral, Thoracic, Transplantation, and Pediatric Surgery, Kurt-Semm Center for Laparoscopic and Robotic Assisted Surgery, University Hospital Schleswig Holstein, Campus Kiel, Arnold Heller Strasse 3, 24105 Kiel, Germany; 2 I. Department of Medicine, University Hospital Schleswig Holstein, Campus Kiel, Arnold Heller Strasse 3, 24105 Kiel, Germany; 3 Department of General, Visceral, Vascular, and Transplantation Surgery, University Hospital Rostock, Schillingallee 35, 18057 Rostock, Germany

**Keywords:** Laparoscopy, Obesity, Robotic surgery, Roux-en-Y gastric bypass

## Abstract

**Background:**

Proximal Roux-en-Y gastric bypass is commonly used to manage obesity, performed using laparoscopic or robot-assisted minimally invasive surgery. As the prevalence of robotic bariatric surgery increases, further data is required to justify its use.

**Methods:**

This was a large, retrospective analysis of prospectively recorded data for Roux-en-Y gastric bypass (RYGB) procedures performed using laparoscopic (LRYGB) or robotic (RRYGB; da Vinci Xi system, Intuitive Surgical Sàrl) surgery between January 2016 and March 2019. The surgical techniques did not differ apart from different trocar placements. Data collected included patient characteristics before and after RYGB, operative outcomes and complications.

**Results:**

In total, 114 RRYGB and 108 LRYGB primary surgeries were performed. There were no significant differences between the groups, apart from a significantly shorter duration of surgery (116.9 vs. 128.9 min, respectively), lower C-reactive protein values at days 1 (31.1 vs. 44.1 mg/l) and 2 (50.3 vs. 77.8 mg/l) after the intervention, and overall complication rate (4.4 vs. 12.0%, Clavien-Dindo classification II-V) with RRYGB compared with LRYGB. There was a lower hemoglobin value in the postoperative course after RRYGB (12.1 vs. 12.6 g/dl, day 2).

**Conclusions:**

In our experience, robotic RYGB has proven to be safe and efficient, with a shorter duration of surgery and lower rate of complications than laparoscopic RYGB. RRYGB is easier to learn and seems safer in less experienced centers. Increasing experience with the robotic system can reduce the duration of surgery over time. Further studies with higher evidence level are necessary to confirm our results.

**Electronic supplementary material:**

The online version of this article (10.1007/s11695-020-04508-1) contains supplementary material, which is available to authorized users.

## Introduction

Proximal Roux-en-Y gastric bypass is the most frequently performed bariatric procedure in Europe [1, 2] and is a standard procedure in the surgical treatment of obesity, particularly in the presence of type 2 diabetes or gastroesophageal reflux disease [1, 3]. The laparoscopic technique [4] is well-established and clearly superior to the open procedure [5]. The operation is safe with low complication rates but technically challenging with a relatively flat learning curve of at least 100 [6] to 500 [7] procedures. In the expectation of overcoming the limitations of laparoscopy and shortening the learning curve [8], the surgical robot was implemented in bariatric surgery.

In 1998, a bariatric operation using a surgical robot was performed for the first time for the implantation of a gastric band [9]. The first robot-assisted Roux-en-Y gastric bypass (RRYGB) followed in 2001 [10]. Since then, various studies have shown that RRYGB is safe and efficient but requires longer operating times and higher costs than laparoscopic RYGB (LRYGB) [11, 12]. Other studies reported lower complication rates, a lower number of revision procedures, and a steeper learning curve with RRYGB [8, 13–17], although some studies reported higher complication rates [18–20]. Meta-analyses have confirmed the lower incidence of complications, longer surgery times, and higher costs associated with RRYGB but criticized the relatively low quality of the existing studies and the lack of large randomized controlled trials [21, 22]. US registry data indicate the increasing prevalence of RRYGB, which comprised 5.8% (*n* = 2282) of all RYGB procedures in 2016 (versus 39,425 LRYGB procedures), with comparable complication rates but longer surgery time (138 min) versus LRYGB (108 min) [23]. Despite the increase, it remains controversial whether the use of the robot in bariatric surgery is justified.

We present the largest German series of RRYGB procedures using the da Vinci Xi® system (Intuitive Surgical Sàrl). The aim of our study was to investigate the effectiveness and safety of laparoscopic versus robotic proximal primary RYGB surgery.

## Materials and Methods

**Fig. 1:**
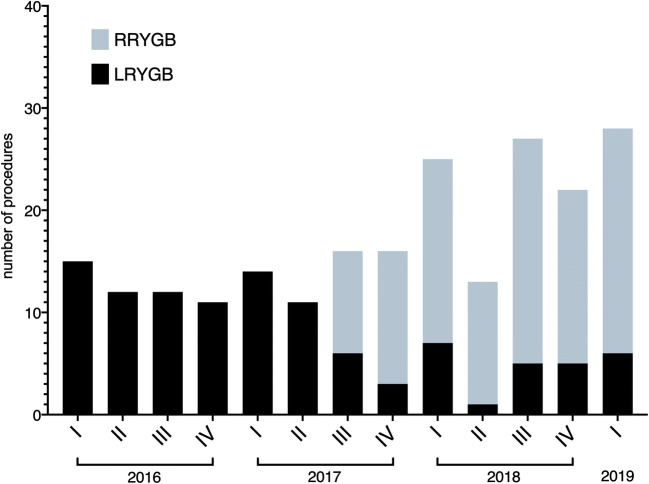
Number of laparoscopic and robotic Roux-en-Y gastric bypass procedures performed per quarter

With the approval of the local ethics committee and provision of written, informed patient consent, all bariatric operations performed at our center have been prospectively recorded since January 2016. The operations were performed by certified bariatric surgeons. At the beginning of 2016, the total experience of the center, among other bariatric and non-bariatric laparoscopic procedures, consisted of 250 LRYGB procedures. Only one surgeon had an experience of over 100 cases. The obesity and metabolic surgery center is certified by the German Society for General and Visceral Surgery (DGAV) since 2015. Furthermore, the department has great expertise in robotic surgery with more than 1000 robot-assisted visceral and thoracic surgical procedures since 2013. The bariatric surgeons performed their first robotic procedures in 2016. Experience with the da Vinci Si® system (Intuitive Surgical Sàrl) was initially gained in sleeve gastrectomy and RYGB surgery [24]. The da Vinci Xi system was first used in August 2017. The surgical indications were based on interdisciplinary recommendations according to current guidelines. In 2016, only laparoscopic surgery was performed. From mid-2017, the surgical procedure was selected according to availability (Fig. 1). Otherwise, there were no specific selection criteria for the use of the da Vinci Xi robot.

From January 2016 to March 2019, all laparoscopic or robotic (da Vinci Xi) primary proximal RYGB surgeries were retrospectively evaluated. In addition to the inpatient course, standardized follow-up data were collected after 1 month and 1 year. A subgroup consisting of 37 primary da Vinci Xi RYGB surgeries was published as single surgeon case series in 2019 [25].

### Surgical and Anastomosis Technique

Laparoscopic and robot-assisted operations were performed in French position. The first access was carried out using a 12-mm FIOS First Entry Trocar (Applied Medical). Laparoscopically, we used five 12-mm trocars; robotically, we used two 12-mm and four 8-mm trocars. Apart from the different trocar placements, the surgical techniques did not differ. The detailed procedure steps have been published previously [25]. In brief, a side-to-side gastrojejunostomy was performed using a 45-mm linear stapler (Echelon Flex™ Endopath). The anastomosis was completed by a running seromuscular suture with resorbable material such as Vicryl or Stratafix. The jejunojejunostomy was performed in the same manner. The biliopancreatic limb was measured 100 cm and the alimentary limb 150 cm, respectively. Perioperative treatment with a single-dose of antibiotics and pneumatic pumps was identical in both groups, according to the local standard.

### Data Acquisition and Statistics

A retrospective analysis of prospectively collected data after laparoscopic and robotic da Vinci Xi gastric bypass surgery was performed to compare the groups with respect to sex, age, weight, body mass index (BMI), Edmonton obesity staging system (EOSS) [26], preoperative hemoglobin, leukocytes, and C-reactive protein (CRP). Postoperative complications within 30 days after surgery were recorded, classified according to the Clavien-Dindo classification [27]. In addition, the estimated blood loss, duration of surgery, laboratory parameters (e.g., leukocytes, hemoglobin loss, CRP after 1 and 2 days), duration of hospital stay, and weight course after 30 days and 12 months were evaluated. The statistical analyses were performed using SPSS 24 (SPSS Inc., Chicago, IL, USA). Continuous parameters were compared by a two-sided *t* test, categorical parameters by a χ2-test. A *p* value < 0.05 was regarded as statistically significant. Values are presented as mean ± standard deviation (SD) or *n* (%), as appropriate.

## Results

### Patient Characteristics

In total, 108 primary LRYGB and 114 primary RRYGB surgeries were performed between 2016 and 2019. Patient demographic data showed no significant differences between the groups, apart from a different distribution with respect to EOSS with a slight increase in comorbidity rates in the robotic group (Table 1).

**Table 1:** Patient characteristics in laparoscopic and robotic Roux-en-Y gastric bypass (RYGB) groups

	Laparoscopic RYGB (*n* = 108)	Robotic RYGB (*n* = 114)	*p* value*
Age (years)	42.7 ± 9.4	42.0 ± 11.3	0.657
Sex (f/m), n (%)	85/23 (78.7/21.3)	86/28 (75.4/24.6)	0.563
Weight (kg)	142.2 ± 20.2	139.3 ± 23.5	0.324
Height (cm)	172.3 ± 9.4	172.3 ± 10.1	0.955
BMI (kg/m^2^)	47.8 ± 4.5	46.7 ± 5.0	0.077
EOSS, n (%)			
I	18 (16.7)	10 (8.8)	
II	45 (41.7)	52 (45.6)	
III	43 (39.8)	42 (36.8)	
IV	2 (1.9)	10 (8.8)	0.046
Hemoglobin (g/dl)	14.3 ± 1.3	13.9 ± 1.4	0.062
Leukocytes (10^9^/l)	8.2 ± 2.0	8.1 ± 2.0	0.519
CRP (mg/l)	10.3 ± 8.9	8.8 ± 6.9	0.160

Values are presented as mean ± standard deviation, unless indicated. *Continuous parameters were compared using the 2-sided *t* test, categorical parameters using the χ2-test. CRP, C-reactive protein; EOSS, Edmonton obesity staging system

### Peri- and Postoperative Results

The duration of surgery with RRYGB (116.9 min) was significantly shorter than with LRYGB (128.9 min), with an average docking time of 6.4 min (Table 2). Intraoperative blood loss was minimal in both groups. Postoperative laboratory results showed decreased hemoglobin values after RRYGB on days 1 and 2. Leukocytes showed no significant differences. Postoperative CRP values were significantly lower after RRYGB. The length of stay did not differ significantly between the two groups (Table 2).

**Table 2:** Operative parameters and 30-day complication rates between laparoscopic and robotic Roux-en-Y gastric bypass (RYGB)

	*n*^*a*^	Laparoscopic RYGB	Robotic RYGB	*p* value*
Operative time (min)	108/114	128.9 ± 34.1	116.9 ± 34.2	0.010
Docking time (min)	—/108	–	6.4 ± 4.1	–
Intraoperative blood loss (ml)	41/86	7.8 ± 13.0	5.9 ± 8.0	0.320
Hemoglobin (g/dl)				
Day 1	107/112	12.7 ± 1.4	12.3 ± 1.3	0.035
Day 2	102/108	12.6 ± 1.5	12.1 ± 1.6	0.024
Leukocytes (10^9^/l)				
Day 1	107/112	10.5 ± 2.9	10.7 ± 2.6	0.536
Day 2	102/108	9.3 ± 2.6	8.7 ± 2.4	0.108
CRP (mg/l)				
Day 1	107/109	44.1 ± 34.4	31.1 ± 14.0	0.0004
Day 2	103/109	77.8 ± 66.1	50.3 ± 29.6	0.0002
Length of stay (days)	108/114	5.6 ± 7.6	4.3 ± 3.5	0.082
Clavien-Dindo classification, *n* (%)	108/114			
0		85 (78.7)	104 (91.2)	
I		10 (9.3)	5 (4.4)	
II		5 (4.6)	2 (1.8)	
IIIa		4 (3.7)	1 (0.9)	
IIIb		3 (2.8)	1 (0.9)	
IVa		0 (0)	1 (0.9)	
IVb		1 (0.9)	0 (0)	
V		0 (0)	0 (0)	0.147
Clavien-Dindo II-V, *n* (%)	108/114	13 (12.0)	5 (4.4)	0.037
Wound infection, *n* (%)	108/114	2 (1.9)	0 (0)	0.144
Hemorrhage, *n* (%)	108/114	2^*b*^ (1.9)	1^*c*^ (0.9)	0.530
Leakage, *n* (%)	108/114	4 (3.7)	1 (0.9)	0.156
Stenosis, *n* (%)	108/114	0 (0)	0 (0)	
Reoperations, *n* (%)	108/114	4 ^*d*^ (3.7)	2^*e*^ (1.8)	0.371
Excess weight loss (%)				
30 days	108/114	20.3 ± 6.8	20.9 ± 7.4	0.575
1 year	92/68	71.3 ± 19.9	72.9 ± 18.2	0.611
BMI change (kg/m^2^)				
30 days	108/114	− 4.5 ± 1.2	− 4.4 ± 1.4	0.501
1 year	92/68	− 16.0 ± 4.4	− 15.2 ± 3.5	0.228

Values are presented as mean ± standard deviation, unless indicated. *Continuous parameters were compared using the 2-sided *t* test, categorical parameters using the χ2-test; a *p* value < 0.05 was considered statistically significant (in bold). ^*a*^laparoscopic/robotic RYGB groups; ^*b*^one gastrointestinal bleeding, one abdominal bleeding; ^*c*^one GI gastrointestinal bleeding; ^*d*^three leakages, one omental necrosis; ^*e*^one omental necrosis, one leakage

In total, 13 (12.0%) complications occurred in LRYGB and 5 (4.4%) in RRYGB, according to the Clavien-Dindo classification II-V. After LRYGB, four revision operations were necessary, one with partial omentum necrosis and three with leakage. Following the necessary revision operation, one patient suffered a heart attack and had to be treated in an intensive care unit. The patient was discharged to a rehabilitation facility after 77 days. The three other patients were all discharged home within 30 days. In addition, one leak of the gastrojejunostomy was healed using endoscopic vacuum therapy only. Intraabdominal hemorrhage was found parallel to leakage of the gastrojejunostomy during revision surgery. One gastrointestinal bleeding was stopped endoscopically. Two superficial wound infections were confirmed postoperatively. No stenoses occurred with LRYGB. Five more patients received postoperative antibiotic therapy; in one case, a urinary tract infection was diagnosed, but no infection was detected in the other four cases.

After RRYGB, two revision operations were performed due to partial omentum necrosis and a leakage of the gastrojejunostomy. The first patient could be discharged 8 days postoperatively; the second patient initially had to be treated in the intensive care unit and was discharged home after 40 days. One patient was endoscopied with gastrointestinal bleeding; an active bleeding source was not found, and an endoscopic intervention or transfusion was not necessary. Wound infections and stenoses did not occur with RRYGB. Two more patients received postoperative antibiotic therapy, and a urinary tract infection was diagnosed in one of these cases.

The weight loss after 30 days and 1 year, measured as percentage excess weight loss and BMI difference, was comparable in both groups (Table 2).

An overview of all patients including Clavien-Dindo classification, LOS, weight loss, and laboratory values is available as supplementary material.

### Learning Curves

**Fig. 2:**
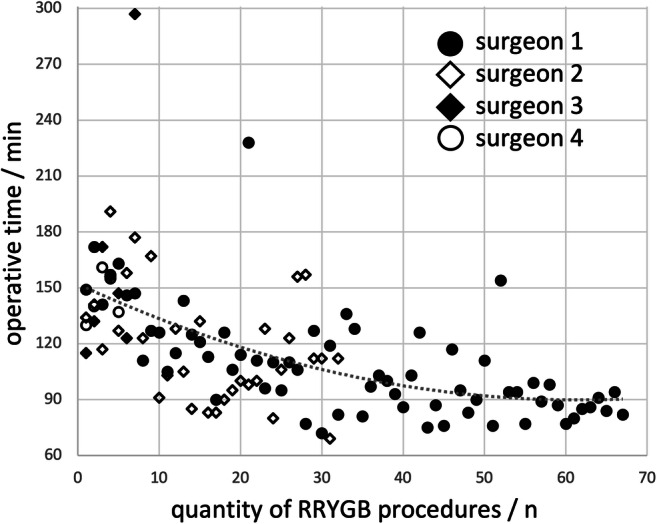
Learning curves of the robotic console surgeons. Surgeons 1 (*n* = 67 procedures) and 2 (*n* = 32) started with da Vinci Xi Roux-en-Y gastric bypass (RYGB) procedures in mid-2017, while Surgeon 3 (*n* = 7) started in June 2018 and Surgeon 4 (*n* = 5) in September 2018

The learning curves of the da Vinci Xi RYGB procedures for the participating surgeons are shown in Fig. 2. With increasing experience, the mean operative time was reduced from 150 min at the beginning to 90 min at the end (range 69–297 min). The major complications observed (Clavien-Dindo 3+) all occurred within the first 20 procedures (Supplementary Material).

## Discussion

In our experience, the use of a robot in primary RYGB surgery is safe and efficient, shortens operative time, and significantly reduces the incidence of overall complications. Considering individual complications such as reoperation, leakage, bleeding, and stenosis, no significant difference could be found due to the low incidence of these events.

With regard to complications, our results confirm other original papers [13, 15, 17, 28] and meta-analysis [21, 22]. Economopoulos found fewer reoperations and strictures after using the robot [21]. Our own analysis indicates fewer revisions required after RRYGB in accordance. The meta-analysis of Li reports a significant reduction of the incidence of anastomotic leak with robotic bariatric surgery [22], again reflecting our findings. In contrast, Benizri et al. observed a higher complication rate when using the robot [18] but compared a robotic surgeon performing a hand anastomosis against a laparoscopic surgeon carrying out a linear stapler anastomosis. Moon et al. also reported high complication rates performing a robot-assisted hand-sewn anastomosis [19]. The observed leaks all occurred at the superior portion of the pouch, which was created laparoscopically before performing a hand-sewn anastomosis with the robot. In contrast, we use an identical anastomosis technique in LRYGB and RRYGB and a group of bariatric surgeons who performed both procedures. Current evaluations of the US registry data show no significant differences regarding complications at higher readmission rates after robotic surgery compared to laparoscopic surgery [29, 30]. The differences between meta-analyses and registry studies may be due to the learning curves. Registry studies should capture all procedures in an unfiltered manner. Thus, as in our study, the initial experiences are also illustrated. In contrast to Sanchez, Senellart and our own study [8, 16], original papers tend to report on later experiences; in meta-analyses, this generally represents a later part of the learning curve. Accordingly, in 2018, Lundberg stated that “Robotic gastric bypass is getting better” after evaluating the 2016 data from the Metabolic and Bariatric Surgery Accreditation and Quality Improvement Program (MBSAQIP) [23].

The operation times for RRYGB were significantly shorter than LRYGB, despite inclusion of the first procedures using the da Vinci Xi system. In contrast to our data, most studies report a longer operative time when using the robot [11, 15, 16, 21–23, 29]. However, the operative time varies considerably between these publications, decreasing from a high of 245 min [13] to 108 min [16]. Although the RRYGB operative time was relatively short in the Senellart study, a loss of time by performing a robotic hand-sewn anastomosis was still reported [16]. Our operative times for RRYGB (116.9 min) were in the lower range reported by other studies. Possible positive influences at our center include structured training with the Xi system and an identical surgical technique for linear anastomosis. A generally flat laparoscopic learning curve may be a possible negative influence on the laparoscopic operative times. None of the surgeons involved in our study came even close to a surgical rate of more than 500 laparoscopic RYGB. Yet according to Doumouras et al., it is only after 500 RYGB procedures that stable low operating times are found in LRYGB [7]. Only one surgeon had an experience of more than 100 LRYGB procedures at the beginning of the study. In addition, the laparoscopic operative times and complication rates were found to be in the upper range compared to other publications [7, 16, 17, 20]. Our learning curves with the da Vinci Xi system show stable operative times of less than 2 h after 20–30 operations. In 2005, Sanchez described a steeper learning curve with the surgical robot compared to conventional laparoscopy [8].

In our study, there was a significantly lower CRP value on days 1 and 2 after RRYGB. We regard this as a possible consequence of a more atraumatic and precise surgical approach. However, there is no evidence in this respect yet. Consecutively, this would also explain lower general complication rates. On the other hand, we found significantly lowered hemoglobin values on days 1 and 2. After calculating the hemoglobin difference compared to the preoperative value, no significant difference was found. There was no clinical correlation. Accordingly, we do not consider the observed differences to be relevant, but further vigilance is recommended.

The duration of the hospital stay after the operation was not significantly influenced by type of surgery. The observed shortening of the inpatient stay by 1.3 days on average after RRYGB is rather a consequence of the temporal divergence of the two cohorts. Compared to other publications, inpatient stays in our center were longer in both groups, which are primarily explained by the national peculiarities of patient care and billing that generally result in longer inpatient stays for bariatric patients in Germany.

Laparoscopically as well as robotically, various anastomotic techniques are applied. We used the linear stapler anastomosis rather than the circular stapler anastomosis or one of a complete “hand”-sewn suture. The linear stapler anastomosis is widely used and is considered to be superior to the circular stapler anastomosis with regard to stenosis rates, wound infections, and operative time [31]. No difference was found with regard to leak rates. The hand-sewn anastomosis compared to the circular stapler anastomosis results in lower wound infection rates and lower gastrointestinal bleeding rates, within the same operative time and comparable safety [32]. Whether these statements are also valid for RRYGB remains to be shown. While most workgroups opted for a robotic hand-sewn anastomosis [8, 11, 12, 15–19, 33], we kept to the well-established technique using the linear stapler. We decided to use an external linear stapler operated by the assistant. It is also feasible to use a robotically controlled linear stapler [34], for which a 12-mm da Vinci trocar is needed and has to be placed somewhat different to achieve a sufficient distance to the target organ.

Costs are important when evaluating whether the robot has advantages or disadvantages in RYGB. There is no doubt that the use of the robot is initially associated with higher costs. We cannot provide a complete cost calculation but assume additional costs for system maintenance, sterile draping, trocars, and instruments of €2000 [35]. With our current reduction in operative times with RRYGB and the corresponding deduction of a minimum of 30 min × €15/min, we assume additional costs of approx. €1500 per RRYGB case. It remains questionable whether these costs are economically profitable by avoiding complications, which has been postulated [14]. It also remains questionable whether advertising effects will recoup the economic costs, which has also been suggested [16]. To reduce costs effectively, an interdisciplinary setting with the highest possible utilization of the system is required, to minimize the high maintenance costs [36].

A possible weakness of the present paper lies in the selection type and in the temporal divergence of the two groups. While laparoscopic procedures were mainly performed between 2016 and 2017, most of the robotic operations took place around 2018. The cohorts are largely comparable. The differences observed in EOSS tended to favor the LRYGB group. The operations were performed by various surgeons, all of whom already had bariatric experience. At the beginning of the study, most of the bariatric surgeons involved were still within the learning curve of an LRYGB as a possible explanation for increased complication rates and operative times. Thus, the conclusions of this paper are valid for bariatric surgeons with limited laparoscopic RYGB experience. The experience with the da Vinci system was limited to bariatric procedures with the Si system before da Vinci Xi was introduced. The entire learning curve with the Xi System can be found in the robotic group. While the latter would benefit the laparoscopic cohort, the overall experience with the procedure gained in laparoscopic RYGB would favor the robotic cohort. The strength of the study lies in the comparable surgical techniques using linear stapler anastomoses. The study provides a detailed picture with a complete 30-day follow-up rate.

## Conclusion

In our experience, robotic RYGB has proven to be safe and efficient. In case of still limited expertise with laparoscopic RYGB procedures, using a robotic system may result in lower complication rates and shorter operative times than laparoscopic RYGB. Whether the benefits outweigh the additional costs required for RRYGB remains to be evaluated. Further studies with higher evidence level are necessary to confirm our results.

## Electronic Supplementary Material

ESM 1 (PDF 494 kb)
