# Supplementary material for: The Impact of Robotics in Learning Roux-en-Y Gastric Bypass: a Retrospective Analysis of 214 Laparoscopic and Robotic Procedures: Robotic Vs. Laparoscopic RYGB
Source: Obes Surg. 2020 Mar 2;30(6):2403–10. doi: 10.1007/s11695-020-04508-1 (PMC7475058; doi:10.1007/s11695-020-04508-1)
Supplement: Supplementary file 1 — (PDF 494 kb) [file 11695_2020_4508_MOESM1_ESM.pdf]

Nr	gender	age	BMI	EOSS	procedure	operative time / min	ClavienDindo30d	LOS / d	%EWL 30d	BMI change 30d	%EWL 1 Year	BMI change 1 Year	Hb preoperative	Hb 1d	Hb 2d	Leucocytes preoperative	Leucocytes 1d	Leucocytes 2d	CRP preoperative	CRP 1d	CRP 2d	Docking / min
1	m	32	43,1	2	LRYGB	115	0	3	34,0%	-6,2	105,9%	-19,2	15,10	13,50	13,10	9,44	12,09	10,26	5,39	46,80	59,50	
2	w	38	42,5	2	LRYGB	126	I	4	24,9%	-4,4	118,5%	-20,7	13,70	13,00	13,40	10,21	11,70	10,55	7,04	133,00	134,00	
3	w	37	55,6	2	LRYGB	133	I	4	16,4%	-5,0	72,7%	-22,2	14,50	11,80	13,00	9,66	8,63	7,99	10,40	140,00	128,00	
4	m	44	42,4	3	LRYGB	123	0	3	41,6%	-7,2	98,6%	-17,2	16,20	14,80	15,40	7,20	8,43	7,67	0,55	16,60	25,00	
5	m	49	50,5	3	LRYGB	120	0	4	6,8%	-1,7	38,0%	-9,7	11,80	12,00	11,80	5,96	9,24	7,26	1,12	14,00	48,00	
6	m	39	52,3	2	LRYGB	160	0	3	10,3%	-2,8	33,1%	-9,1	15,20	13,50	13,60	8,73	13,20	10,15	18,40	87,10	110,00	
7	m	35	51,5	1	LRYGB	125	IIIa	7	16,8%	-4,5	43,0%	-11,4	15,60	14,00		4,67	7,70		3,73	23,80		
8	w	44	53,4	3	LRYGB	106	0	5	15,5%	-4,4	45,2%	-12,8	12,00	10,10	10,70	13,00	11,22	9,83	50,60	111,00	101,00	
9	m	53	53,7	3	LRYGB	150	0	4	24,2%	-6,9	62,0%	-17,8	16,60	15,30	15,30	8,20	9,71	10,47	7,24	58,20	152,00	
10	w	51	43,8	3	LRYGB	112	0	4	21,9%	-4,1	72,9%	-13,7	15,30	13,10	12,80	9,21	9,12	14,81	3,80	11,70	134,00	
11	w	34	50,7	2	LRYGB	203	II	7	16,6%	-4,3	70,4%	-18,1	14,50	13,90	12,60	11,59	20,63	17,37	5,91	107,00	263,00	
12	w	52	47,3	2	LRYGB	111	0	5	27,7%	-6,2	77,5%	-17,3	15,30	12,90	13,70	9,05	10,69	9,75	10,40	24,70	39,90	
13	w	60	49,7	3	LRYGB	169	0	5	23,3%	-5,7	65,7%	-16,2	14,50	11,70	12,40	9,69	11,58	10,24	9,39	35,80	62,60	
14	w	45	50,7	3	LRYGB	120	0	5	15,1%	-3,9	96,8%	-24,9	12,90	12,00	12,30	8,37	13,06	13,39	6,98	45,00	132,00	
15	w	44	46,7	1	LRYGB	116	0	5	15,6%	-3,4	56,2%	-12,2	11,70	10,50	10,50	6,05	8,39	6,29	17,80	33,50	61,80	
16	w	46	48,8	3	LRYGB	135	0	5	15,5%	-3,7	67,4%	-16,0	12,50	10,50	9,50	5,10	9,28	7,66	10,00	32,70	44,00	
17	w	48	46,3	2	LRYGB	127	0	4	30,0%	-6,4	77,8%	-16,6	13,80	11,10	10,00	4,36	5,98	4,66	2,50	27,60	46,80	
18	w	35	41,3	3	LRYGB	177	0	4	22,3%	-3,6	123,7%	-20,1	14,10	12,70	11,80	6,44	8,48	7,36	1,32	17,90	22,30	
19	w	52	46,5	2	LRYGB	150	0	4	31,2%	-6,7	79,6%	-17,1	12,90	12,30	10,30	14,31	11,21	9,58	12,00	67,70	144,00	
20	w	53	51,9	3	LRYGB	320	IVb	77	23,4%	-6,3	65,2%	-17,5	15,80	15,80	15,40	8,78	12,62	11,68	20,80	118,00	147,00	
21	w	49	46,1	3	LRYGB	101	0	6	20,4%	-4,3	61,1%	-12,9	15,90	12,70	13,00	9,66	15,23	9,34	27,60	48,00	46,60	
22	w	43	46,0	2	LRYGB	153	0	6	23,3%	-4,9	79,1%	-16,7	13,00	11,10	10,70	7,45	11,49	9,67	14,30	41,80	93,10	
23	w	68	43,3	3	LRYGB	103	IIIb	22	18,5%	-3,4	37,1%	-6,8	15,50	12,50	13,10	9,90	10,33	15,10	14,50	111,00	408,00	
24	w	57	40,1	3	LRYGB	134	0	3	28,6%	-4,3	71,4%	-10,8	14,80	12,80	12,50	7,57	8,03	6,96	10,50	40,60	56,70	
25	w	33	52,1	2	LRYGB	134	0	3	17,3%	-4,7	95,5%	-25,9	12,00	11,20	10,60	6,04	5,62	4,86	6,25	25,60	23,40	
26	w	47	39,1	1	LRYGB	118	0	6	31,9%	-4,5	77,2%	-10,9	14,80	12,90	13,30	7,08	9,46	8,55	6,57	27,10	40,30	
27	w	38	51,5	3	LRYGB	114	0	4	24,9%	-6,6	51,1%	-13,5	10,10	8,80	9,30	6,39	6,85	5,35	6,46	32,00	43,50	
28	w	53	41,3	3	LRYGB	196	IIIb	17	30,0%	-4,9	40,8%	-6,7	16,00	13,60	14,70	7,80	9,94	16,34	14,50	99,20	297,00	
29	w	39	47,9	3	LRYGB	137	I	5	20,9%	-4,8	86,7%	-19,8	13,30	12,00	10,90	6,85	10,93	8,63	3,73	68,50	181,00	
30	w	30	47,7	1	LRYGB	160	II	5	7,5%	-1,7	59,7%	-13,5	14,10	12,10	12,80	11,73	12,46	13,97	23,70	59,30	133,00	
31	w	49	46,3	3	LRYGB	138	IIIa	4	16,9%	-3,6	84,3%	-17,9	13,60	12,30	12,40	9,44	14,24	11,52	16,10	33,80	45,50	
32	w	46	46,8	3	LRYGB	113	0	4	18,4%	-4,0	92,1%	-20,0	14,90	13,80	12,50	10,55	13,28	8,70	21,60	24,30	41,80	
33	w	50	46,0	2	LRYGB	106	II	10	19,8%	-4,1	77,7%	-16,3	13,70	10,40	10,60	7,96	7,73	8,62	3,20	31,00	39,10	
34	w	46	42,9	2	LRYGB	110	0	4	18,9%	-3,4	84,8%	-15,2	14,30	13,00	13,40	6,64	9,32	9,07	3,41	25,10	42,60	
35	w	47	48,9	1	LRYGB	145	I	4	19,3%	-4,6			15,20	13,10	13,40	7,75	6,98	6,48	10,60	85,70	123,00	
36	w	24	54,6	1	LRYGB	150	0	4	14,1%	-4,2	56,4%	-16,7	12,70	11,70	10,70	5,71	8,26	6,08	3,91	36,40	68,20	
37	w	41	45,4	2	LRYGB	134	0	5	24,4%	-5,0			14,10	13,30	12,80	7,30	10,50	9,98	6,73	42,80	86,60	
38	w	40	43,3	3	LRYGB	103	IIIb	28	32,5%	-5,9	74,1%	-13,5	12,20	11,80	10,90	10,34	16,12	14,50	27,00	255,00	353,00	
39	w	36	54,3	2	LRYGB	125	0	5	16,5%	-4,8	88,5%	-26,0	12,60	10,50	10,60	7,24	10,05	8,82	12,10	50,20	121,00	
40	w	37	44,3	1	LRYGB	175	0	4	23,9%	-4,6	106,5%	-20,5	15,00	12,50	12,60	4,85	6,97	6,85	0,70	35,60	70,00	
41	w	48	51,3	3	LRYGB	124	II	5	17,0%	-4,5	55,1%	-14,5	13,00	11,90	12,00	9,35	8,21	8,30	31,40	35,60	121,00	
42	w	45	42,4	3	LRYGB	121	0	4	18,1%	-3,2	54,4%	-9,5	13,70	12,90	13,10	8,10	12,44	10,28	8,37	17,10	18,40	
43	w	48	43,9	3	LRYGB	122	0	5	23,2%	-4,4	92,8%	-17,6	15,10	13,10	13,50	6,45	6,91	6,37	2,98	27,70	32,40	
44	w	45	49,1	2	LRYGB	176	0	5	17,8%	-4,3	74,3%	-17,9	15,30	14,10	14,10	7,00	7,65	7,43	6,88	55,90	104,00	
45	w	34	47,1	2	LRYGB	95	0	4	26,9%	-5,9			14,80	13,60	13,50	8,80	9,21	9,57	6,42	27,40	92,00	
46	w	41	48,9	3	LRYGB	133	0	4	15,7%	-3,8	51,9%	-12,4	13,40	10,60	11,70	9,70	7,50	8,45	14,00	64,50	84,00	
47	w	33	48,7	2	LRYGB	98	0	3	13,7%	-3,2	82,2%	-19,5	14,90	12,40	13,70	8,11	7,39	8,02	15,60	42,60	55,10	
48	w	56	49,7	3	LRYGB	118	0	3	15,0%	-3,7	41,3%	-10,2	15,50	13,90	13,80	6,39	8,87	7,69	1,90	34,80	53,40	
49	w	30	46,0	2	LRYGB	108	0	3	23,5%	-4,9			10,60	9,70	9,20	9,69	15,96	10,76	4,25	11,80	11,60	
50	w	44	44,6	3	LRYGB	162	0	4	20,3%	-4,0			13,40	12,00	12,70	8,82	11,63	10,83	3,37	15,60	27,80	
51	w	51	51,9	2	LRYGB	141	0	5	12,3%	-3,3	43,0%	-11,6	15,30	11,80	12,20	8,58	9,92	7,83	41,40	51,30	56,50	
52	w	41	43,4	3	LRYGB	124	0	4	19,5%	-3,6	62,0%	-11,4	13,50	12,90	13,30	9,06	13,53	10,90	4,81	23,10	39,40	
53	w	36	39,7	2	LRYGB	92	0	5	43,7%	-6,4	112,1%	-16,4	14,30	12,30	12,60	8,09	8,99	7,55	12,30	52,50	94,10	
54	w	48	51,3	3	LRYGB	116	0	5	9,9%	-2,6	48,0%	-12,6	15,40	13,80	13,50	11,48	17,70	12,69	15,50	26,60	61,90	
55	w	34	48,9	2	LRYGB	108	0	4	12,6%	-3,0			13,20	12,10	11,70	9,92	11,75	11,72	16,70	29,00	63,70	
56	w	36	56,8	2	LRYGB	124	I	5	10,8%	-3,4	42,0%	-13,3	13,10	11,80		10,40	10,71		26,00	62,50	120,00	
57	w	40	43,9	2	LRYGB	103	0	4	19,2%	-3,6	89,0%	-16,8	13,80	12,60		6,16	9,43		2,67	19,30		
58	w	65	50,8	3	LRYGB	118	IIIa	5	29,8%	-7,7	46,9%	-12,1	14,50	10,40	9,20	6,72	7,96	6,33	3,56	23,30	49,00	
59	w	51	46,2	3	LRYGB	122	I	4	23,8%	-5,0	37,5%	-8,0	14,20	14,20	13,50	11,06	16,45	12,68	6,69	31,10	108,00	
60	w	30	46,1	2	LRYGB	104	0	4	18,0%	-3,8	101,8%	-21,5	14,70	13,00	12,50	7,66	9,02	7,63	3,49	19,20	36,50	
61	w	53	48,5	1	LRYGB	192	0	4	23,9%	-5,6	102,4%	-24,0	15,50	13,70	13,60	7,70	9,01	9,44	6,15	31,30	69,90	
62	w	57	42,6	4	LRYGB	94	0	5	28,9%	-5,1	75,6%	-13,3	13,50	11,40	12,00	4,87	5,41	4,96	5,01	18,90	38,40	
63	w	42	55,1	1	LRYGB	85	0	4	14,6%	-4,4	79,3%	-23,9	14,30	12,80	12,90	9,10	9,63	8,65	12,40	76,70	94,20	
64	w	29	48,0	2	LRYGB	123	0	5	24,8%	-5,7	46,4%	-10,7	14,70	14,70	13,70	5,69	7,45	7,08	4,29	22,40	31,10	
65	w	49	42,0	2	LRYGB	112	0	5	23,6%	-4,0	100,0%	-17,0	13,70	13,10	13,20	5,67	7,57	5,61	3,59	22,60	50,50	
66	w	34	47,5	2	LRYGB	139	0	4	22,1%	-5,0	75,7%	-17,0	14,90	12,60	12,50	5,90	8,01	6,85	7,12	37,60	60,70	
67	w	38	48,0	2	LRYGB	205	0	3	22,0%	-5,0	59,1%	-13,6	16,30	14,20	14,30	5,87	3,63	3,55	6,38	55,60	67,10	
68	w	61	48,9	2	LRYGB	138	0	4	13,2%	-3,2	67,3%	-16,1	12,40	12,10	11,20	6,42	11,19	8,46	5,30	37,40	53,20	
69	w	51	56,1	3	LRYGB	123	0	3	17,8%	-5,5			15,90	13,60	14,10	11,95	11,18	12,14	33,60	51,20	139,00	

																						Docking / min
70	w	42	47,2	3	LRYGB	185	0	5	15,5%	-3,4	55,4%	-12,3	13,90	14,00	14,20	9,00	15,08	12,77	16,90	55,20	92,60	
71	w	54	47,4	2	LRYGB	105	I	5	13,9%	-3,1	63,3%	-14,2	13,20	11,70	11,50	7,67	8,91	8,44	28,00	55,20	70,50	
72	w	45	39,5	2	LRYGB	112	0	4	21,8%	-3,2	98,3%	-14,2	15,00	14,90	11,90	5,02	11,36	7,27	2,38	22,60	28,50	
73	w	35	53,1	2	LRYGB	122	0	4	15,7%	-4,4	75,9%	-21,3	13,80	12,50	13,30	10,40	13,81	12,70	11,20	21,00	19,40	
74	w	21	52,6	1	LRYGB	120	0	4	10,5%	-2,9	39,4%	-10,9	15,20	12,20	12,80	9,82	14,93	9,91	6,32	11,10	23,80	
75	w	32	53,2	1	LRYGB	98	0	4	13,9%	-3,9	55,5%	-15,7	13,80	12,90	12,70	8,76	11,65	10,04	15,10	31,40	45,20	
76	w	33	53,5	3	LRYGB	153	0	4	16,4%	-4,7	80,8%	-23,0	14,80	12,40	12,20	6,94	11,04	8,50	3,78	26,40	24,70	
77	w	57	42,5	2	LRYGB	206	I	5	29,9%	-5,2	74,9%	-13,1	15,00	13,20	12,30	7,53	14,27	11,20	8,22	96,90	147,00	
78	w	32	53,9	2	LRYGB	158	0	4	12,9%	-3,7	64,3%	-18,6	12,10	10,40	10,30	6,78	7,35	8,24	9,79	27,70	61,00	
79	m	45	51,7	2	LRYGB	168	0	4	14,5%	-3,9	72,7%	-19,4	15,70	13,70	13,60	5,54	8,06	6,71	2,21	33,60	56,70	
80	w	36	56,2	2	LRYGB	139	0	4	15,3%	-4,8	65,9%	-20,6	14,20	12,90	13,60	8,39	10,08	11,08	9,82	14,20	18,80	
81	w	39	57,7	3	LRYGB	167	0	4	19,8%	-6,5	51,9%	-17,0	18,20	17,10	16,80	7,77	13,85	11,80	3,77	12,90	58,80	
82	w	43	53,7	3	LRYGB	189	0	5	18,2%	-5,2	83,8%	-24,0	13,10	12,00	11,70	7,53	10,03	8,91	14,50	85,80	117,00	
83	w	48	50,9	2	LRYGB	106	0	4	18,7%	-4,8	68,2%	-17,6	13,70	11,30		12,09	8,13		15,30	22,70		
84	w	44	47,7	2	LRYGB	170	I	5	28,8%	-6,5	87,8%	-19,9	14,80	13,70	12,90	11,54	18,03	16,18	10,00	92,00	207,00	
85	w	33	52,6	2	LRYGB	149	0	4	15,0%	-4,2	90,2%	-24,9	16,60	15,30	14,90	11,25	14,60	11,67	2,80	16,70	14,70	
86	w	39	51,2	1	LRYGB	114	0	4	27,7%	-7,3	70,0%	-18,3	13,40	12,50	11,90	7,76	10,36	8,32	7,52	25,00	61,50	
87	w	31	45,3	1	LRYGB	108	0	4	14,1%	-2,9	59,7%	-12,1	14,00	12,70	13,20	8,83	10,06	7,73	28,00	42,70	64,60	
88	w	57	46,2	2	LRYGB	117	0	4	24,4%	-5,2	62,5%	-13,2	16,00	14,30	14,10	10,09	13,44	9,96	17,60	57,30	61,60	
89	w	26	51,0	2	LRYGB	76	II	7	20,4%	-5,3	69,4%	-18,1	13,10	11,80	12,70	9,59	12,07	12,50	17,20	69,90	109,00	
90	w	33	45,8	3	LRYGB	99	0	4	26,3%	-5,5	92,5%	-19,3	15,10	14,00	14,50	10,77	10,86	10,16	6,66	22,60	39,20	
91	w	62	47,8	4	LRYGB	122	0	4	19,8%	-4,5	70,0%	-15,9	15,00	12,40	12,30	8,28	9,45	7,18	4,61		37,10	
92	w	38	45,9	3	LRYGB	105	0	5	18,2%	-3,8	58,8%	-12,3	15,20	13,50	13,20	5,80	8,44	7,18	3,51	34,00	42,40	
93	w	46	52,5	2	LRYGB	97	0	4	15,6%	-4,3	61,2%	-16,8	15,40	13,10	13,80	9,59	10,91	9,35	2,96	31,90	32,40	
94	w	54	41,5	3	LRYGB	98	0	5	35,7%	-5,9	63,4%	-10,4	16,40	15,60	14,70	9,64	13,54	10,37	5,63	23,80	27,50	
95	w	28	38,0	3	LRYGB	112	0	4	17,8%	-2,3	94,1%	-12,2	14,50	13,40	13,10	9,36	9,49	8,67	9,85	46,40	82,40	
96	w	49	42,1	3	LRYGB	118	I	6	33,2%	-5,7	76,2%	-13,0	13,50	9,80	9,80	10,52	9,82	7,73	6,43	35,40	44,70	
97	w	32	48,3	3	LRYGB	111	0	3	14,0%	-3,3	67,2%	-15,7	15,20	13,00	13,10	6,03	7,70	7,00	7,47	32,40	53,10	
98	w	36	48,6	2	LRYGB	87	0	4	21,8%	-5,1	63,9%	-15,0	14,90	13,70	15,50	6,97	9,30	9,24	0,92	8,84	11,50	
99	w	50	40,0	1	LRYGB	87	0	4	18,9%	-2,8	91,9%	-13,8	14,60	12,60		9,84	14,32		6,08	41,30		
100	w	29	47,7	2	LRYGB	86	0	5	13,4%	-3,0			11,70	10,70	10,10	7,17	6,42	4,75	15,20	22,30	15,80	
101	w	34	44,6	1	LRYGB	108	0	4	18,0%	-3,5			13,80	11,20	10,90	7,58	10,57	10,47	2,41	29,40	40,70	
102	w	38	49,6	1	LRYGB	118	0	5	13,3%	-3,3			12,90	10,90		6,22	12,03		1,55	14,70		
103	w	35	45,5	2	LRYGB	102	0	3	20,5%	-4,2			14,90	12,80	12,90	5,39	9,00	7,08	1,99	19,70	44,60	
104	w	44	41,3	3	LRYGB	119	0	3	25,0%	-4,1			16,30	14,50	15,00	8,40	8,66	8,10	1,86	64,30	104,00	
105	w	53	47,0	3	LRYGB	103	0	4	20,0%	-4,4			14,40		13,30	9,06		9,35	10,60	38,00	60,10	
106	w	29	55,3	1	LRYGB	93	0	4	11,9%	-3,6			12,40	10,70	10,30	9,38	10,72	9,27	23,80	21,90	22,20	
107	w	39	49,1	1	LRYGB	104	0	4	22,3%	-5,4			15,20	14,60	14,90	6,60	10,97	10,18	3,25	21,30	30,30	
108	w	52	42,9	3	LRYGB	120	IIIa	4	18,9%	-3,4			14,40	13,6	13,00	8,24	8,88	6,66	13,50	37,70	45,80	
1	w	38	41,4	3	RRYGB	149	0	4	35,5%	-5,8	116,9%	-19,2	14,20	11,70	11,70	9,37	14,02	10,62	5,96	43,80	55,10	22
2	w	51	37,6	2	RRYGB	134	0	4	26,7%	-3,3	91,8%	-11,5	14,10	12,30	13,20	6,72	9,16	11,99	5,13	44,80	106,00	11
3	w	34	51,6	2	RRYGB	141	0	5	14,7%	-3,9	63,5%	-16,9	15,20	14,00	13,10	7,48	11,16	9,14	3,55	49,60	52,20	27
4	m	50	40,0	4	RRYGB	172	IIIa	4	33,8%	-5,1	83,5%	-12,5	16,60	14,50	15,20	8,14	8,39	7,82	4,18	18,00	42,20	10
5	w	33	50,0	2	RRYGB	141	0	4	21,3%	-5,3	92,3%	-23,0	11,60	11,30	10,10	10,88	17,80	12,77	23,80	28,10	76,10	14
6	m	51	45,2	1	RRYGB	117	0	4	19,2%	-3,9	67,4%	-13,6	14,70	13,30	13,70	6,59	7,81	7,07	1,96	21,90	37,40	8
7	w	42	52,3	2	RRYGB	157	0	5	20,1%	-5,5	68,6%	-18,7	14,80	13,10	13,50	6,76	12,87	11,49	3,97	22,10	23,70	11
8	w	45	45,8	3	RRYGB	191	IVa	40	22,5%	-4,7	85,2%	-17,7	14,80	13,70	14,60	9,01	11,84	4,02	6,31	13,20	20,80	11
9	w	31	54,1	3	RRYGB	163	0	5	17,5%	-5,1	58,7%	-17,1	14,10	11,00	11,30	5,72	8,10	7,29	0,30		34,80	16
10	w	54	50,4	3	RRYGB	146	0	4	13,3%	-3,4	34,0%	-8,7	15,00	12,80	11,60	7,34	9,86	8,40	0,88	23,10	20,10	7
11	w	47	53,2	1	RRYGB	127	0	4	19,5%	-5,5	49,6%	-14,0	14,30	13,90	14,00	6,80	8,67	6,91	8,79	24,40	40,70	19
12	w	56	46,8	3	RRYGB	147	0	4	13,3%	-2,9	53,3%	-11,6	14,10	12,80	12,60	7,45	9,72	6,31	8,96	34,20	27,20	13
13	w	40	46,7	2	RRYGB	158	0	3	11,9%	-2,6	61,1%	-13,2	13,90	11,20	10,60	6,83	7,27	5,68	7,85	44,50	47,00	13
14	w	50	51,4	2	RRYGB	111	0	4	24,2%	-6,4	61,8%	-16,3	8,50	8,20	8,00	11,11	12,15	10,47	4,24	39,80	61,80	15
15	w	45	48,5	2	RRYGB	177	0	4	18,1%	-4,3	55,7%	-13,1	11,90	9,50	8,30	11,34	18,00	14,31	4,94	49,60	48,80	8
16	w	51	38,9	2	RRYGB	127	0	2	37,4%	-5,2	106,3%	-14,8	14,80	13,20	12,90	6,44	11,24	8,56	3,89	39,40	53,00	8
17	w	69	44,8	3	RRYGB	126	0	4	29,7%	-5,9	56,3%	-11,1	13,90	12,60	13,00	5,72	9,88	6,49	6,68	30,90	35,70	12
18	w	51	46,9	2	RRYGB	105	I	5	23,2%	-5,1	71,4%	-15,6	13,80	10,30	9,40	8,60	9,02	6,49	6,23	22,40	43,60	8
19	w	51	52,1	2	RRYGB	115	IIIb	8	15,0%	-4,1	51,2%	-13,9	14,60	13,00	13,50	11,05	12,24	14,01	11,80	41,00	208,00	12
20	w	61	44,1	3	RRYGB	123	I	5	17,7%	-3,4	66,1%	-12,7	13,80	12,20	13,80	9,87	10,42	13,18	10,20	46,90	108,00	5
21	w	28	48,7	3	RRYGB	143	0	4	40,1%	-9,5	93,6%	-22,2	13,10	12,20	12,20	7,77	10,60	8,23	10,10		54,00	8
22	w	52	39,8	3	RRYGB	167	0	4	18,4%	-2,7	89,5%	-13,3	15,30		13,40	11,49		12,81	7,12		40,40	6
23	w	34	49,7	3	RRYGB	91	0	4	18,4%	-4,6	76,5%	-18,9	15,10	13,20	13,10	9,21	14,35	10,78	1,95	12,70	14,50	8
24	w	26	49,1	2	RRYGB	141	0	4	21,5%	-5,2	64,5%	-15,6	14,10	11,20	11,20	7,69	10,64	9,02	25,90	49,90	45,00	5
25	w	53	48,4	3	RRYGB	125	0	4	12,6%	-3,0	86,9%	-20,4	15,60	13,90	14,20	5,86	10,40	8,76	2,05	31,50	22,80	14
26	w	41	38,8	4	RRYGB	103	0	4	47,8%	-6,6			11,90	10,30	10,00	12,75	12,70	8,51	22,60	36,40	52,10	6
27	w	53	40,2	3	RRYGB	121	0	4	24,4%	-3,7	95,5%	-14,5	16,30	14,30	14,50	11,56	14,67	12,58	2,87	25,20	45,40	7
28	w	40	48,9	2	RRYGB	107	0	5	14,5%	-3,5			14,00	12,90	11,40	9,52	11,09	7,52	8,50	42,70	86,50	5
29	w	41	40,8	3	RRYGB	128	0	5	28,4%	-4,5	59,0%	-9,3	14,00	11,90	11,50	7,70	8,44	7,65	13,80	37,10	63,50	8
30	w	62	51,3	3	RRYGB	113	0	4	17,2%	-4,5	45,9%	-12,1	16,90	14,90	15,10	9,85	10,21	8,42	8,19	25,30	67,40	6
31	w	52	48,3	3	RRYGB	105	0	4	17,4%	-4,0	60,9%	-14,2	16,50	14,00	14,30	8,00	8,90	8,98	22,20	31,40	86,10	9
32	w	21	44,4	2	RRYGB	85	II	4	18,1%	-3,5	80,8%	-15,6	12,70	11,60	11,30	5,15	6,24	4,48	4,39	6,82	11,40	8
33	w	29	45,3	2	RRYGB	90	0	4	23,7%	-4,8	87,0%	-17,6	12,00	10,10	9,80	8,45	8,64	7,04	8,54	20,00	33,20	6
34	w	31	52,9	2	RRYGB	166	0	4	17,2%	-4,8	69,7%	-19,4	14,40	12,60		7,23	9,85		7,52	25,20	42,60	10
35	w	52	43,3	2	RRYGB	132	0	4	14,7%	-2,7	68,5%	-12,5	14,80	12,30	10,70	7,22	8,80	5,12	6,61	27,30	62,60	6
36	w	64	47,8	4	RRYGB	126	0	5	15,5%	-3,5	57,4%	-13,1	14,00	12,60	13,30	11,32	13,59	13,10	9,01	26,50	74,30	3
37	w	54	47,0	2	RRYGB	106	0	3	13,8%	-3,0			15,30	13,40	14,00	7,91	10,13	9,39	9,11	66,30	81,00	6
38	w	31	44,3	2	RRYGB	114	0	4	25,4%	-4,9			17,00	14,90	16,60	7,68	8,66	8,27	1,35		47,20	6

39	w	37	41,7	3	RRYGB	83	0	5	17,3%	-2,9	99,8%	-16,7	14,40	13,00	12,50	7,33	8,01	6,75	4,97	35,30	40,80	6
40	w	33	40,1	2	RRYGB	83	0	4	13,3%	-2,0	77,5%	-11,7	13,20	12,20	12,50	8,30	10,44	11,80	22,00	54,20	109,00	7
41	w	25	46,6	2	RRYGB	90	0	4	12,2%	-2,6	65,8%	-14,2	11,70	10,50	10,40	5,59	7,23	6,75	8,59	30,90	56,40	6
42	w	52	47,3	3	RRYGB	228	0	5	25,2%	-5,6			14,80	13,20	13,20	8,60	11,41	10,46	3,75	39,50	89,10	4
43	w	53	41,2	2	RRYGB	95	0	4	33,6%	-5,4			12,80	12,20	12,80	8,79	8,96	7,24	16,40	40,30	36,80	3
44	m	39	48,3	2	RRYGB	111	0	4	24,8%	-5,8	86,9%	-20,2	15,90	12,60	11,00	6,94	10,54	10,29	2,16	20,80	41,90	4
45	w	46	47,8	2	RRYGB	96	0	4	16,1%	-3,7	88,8%	-20,2	12,90	12,10	10,90	7,06	13,50	8,39	16,70	17,30	12,50	6
46	w	19	53,3	2	RRYGB	110	0	4	15,6%	-4,4			13,90	12,10	11,60	6,83	8,65	9,18	20,40	58,50	66,50	4
47	w	52	44,3	2	RRYGB	95	0	3	18,8%	-3,6	81,0%	-15,6	14,20	14,00	13,90	6,91	11,12	9,36	6,17	33,70	53,40	5
48	w	23	53,8	2	RRYGB	110	I	4	5,0%	-1,4	58,3%	-16,8	12,80	11,60	11,50	8,88	10,28	8,26	21,40	53,00	82,80	3
49	w	40	45,7	3	RRYGB	106	0	4	25,1%	-5,2	58,6%	-12,1	16,10	13,20	12,70	12,48	14,09	8,89	9,51	17,50	30,60	4
50	w	38	44,0	1	RRYGB	115	0	4	7,9%	-1,5	77,1%	-14,7	13,40	11,90	12,00	7,38	11,70	7,88	4,44	17,60	24,70	4
51	w	21	45,9	1	RRYGB	77	0	4	23,7%	-5,0			13,80	10,90	10,80	8,19	7,67	4,63	12,40	41,30	60,30	3
52	w	60	50,0	4	RRYGB	127	0	5	16,2%	-4,1			14,00	11,50	11,90	10,43	11,30	8,67	19,30	28,00	65,00	9
53	w	33	49,3	3	RRYGB	100	0	5	24,3%	-5,9	93,6%	-22,8	12,40	10,90	12,30	4,94	5,58	6,29	3,99	42,30	44,50	5
54	w	30	46,5	2	RRYGB	72	0	4	20,5%	-4,4	80,2%	-17,2	13,90	12,60	12,60	8,45	9,87	8,70	2,70	24,00	44,90	4
55	w	43	52,1	3	RRYGB	119	0	4	16,2%	-4,4	55,0%	-14,9	14,50	14,00	13,00	8,36	13,87	10,32	2,20	12,50	20,00	6
56	w	47	44,8	1	RRYGB	82	0	4	24,1%	-4,8	80,0%	-15,8	13,70	11,20	11,40	6,78	11,92	6,94	3,40	60,60	57,80	4
57	w	30	45,3	1	RRYGB	136	0	2	26,2%	-5,3	91,8%	-18,7	14,50	12,90	12,80	10,98	13,32	11,32	9,12	42,20	45,20	6
58	w	44	42,6	2	RRYGB	98	0	5	26,9%	-4,7	104,0%	-18,3	13,40	11,40	11,90	9,26	12,40	9,42	6,20	16,70	28,40	5
59	w	49	47,3	3	RRYGB	132	0	3	17,0%	-3,8	63,6%	-14,2	14,00	12,60	11,80	5,11	8,81	7,05	3,97	34,50	20,30	5
60	w	46	48,9	3	RRYGB	128	0	4	20,6%	-4,9	72,2%	-17,3	13,40	12,70	12,90	8,05	13,45	9,03	3,61	19,70	19,50	7
61	w	43	38,8	4	RRYGB	81	0	5	19,4%	-2,7	80,1%	-11,0	13,00	12,10	11,80	10,16	12,91	7,13	2,59	12,30	31,80	4
62	w	67	51,3	3	RRYGB	172	0	4	15,5%	-4,1	50,9%	-13,4	13,20	12,60		9,84	13,89		12,10	55,90		7
63	w	40	42,2	3	RRYGB	97	0	4	28,5%	-4,9			12,60	12,40	12,50	11,14	18,08	10,89	2,50	7,24	18,60	8
64	w	47	45,0	3	RRYGB	100	0	4	23,4%	-4,7	55,9%	-11,2	16,70	16,00	16,70	9,10	11,44	14,26	10,00	29,60	96,70	7
65	w	21	52,0	1	RRYGB	156	0	4	17,7%	-4,8	60,8%	-16,4	13,40	12,20	11,70	8,18	11,52	8,47	16,60	35,30	29,10	4
66	w	59	37,6	2	RRYGB	147	0	4	14,1%	-1,8	110,0%	-13,8	13,30	11,80	12,40	4,74	7,31	6,75	47,40	30,40	72,80	
67	w	45	43,3	3	RRYGB	123	0	5	32,9%	-6,0	92,6%	-16,9	13,30	10,80	11,20	6,53	9,03	7,56	5,02	25,30	47,60	6
68	w	24	57,0	2	RRYGB	128	I	4	20,7%	-6,6			15,00	12,30	12,30	5,45	9,50	7,90	8,88	94,50	138,00	1
69	w	44	44,6	3	RRYGB	297	0	5	15,9%	-3,1	51,1%	-10,0	14,90	12,40	13,00	7,16	11,40	7,73	14,70	36,30	43,70	
70	w	52	36,2	3	RRYGB	80	0	5	18,6%	-2,1	59,4%	-6,7	13,70	13,90	11,70	7,13	10,97	5,40	7,95	25,80	28,40	
71	w	41	46,0	2	RRYGB	106	0	4	21,4%	-4,5	90,5%	-19,0	13,50	11,90	11,90	8,09	11,47	10,35	1,98	34,80	35,90	
72	w	44	48,8	3	RRYGB	123	0	4	25,6%	-6,1	80,1%	-19,1	12,50	11,60	11,60	8,89	15,52	10,72	4,20	21,70	47,10	4
73	w	42	43,0	3	RRYGB	103	0	4	29,9%	-5,4			16,30	13,20	12,30	14,53	15,74	13,22	3,21	33,60	42,10	4
74	w	39	46,1	2	RRYGB	100	0	4	20,7%	-4,4			13,90	12,60	11,80	6,01	10,41	6,75	10,70	25,30	37,30	3
75	w	27	43,6	2	RRYGB	130	0	3	23,0%	-4,3	78,2%	-14,5	12,40	10,80	11,50	6,87	11,11	10,10	1,34	12,70	47,30	
76	w	41	47,3	2	RRYGB	93	0	3	21,2%	-4,7			15,40	12,40	13,40	5,50	9,71	7,92	2,10	20,10	41,30	5
77	w	42	45,5	1	RRYGB	156	0	4	26,7%	-5,5	91,3%	-18,7	10,80	8,80	8,40	6,79	9,83	7,67	6,91	20,60	31,50	5
78	w	38	46,0	2	RRYGB	86	0	4	18,3%	-3,8	68,5%	-14,4	13,60	10,40	9,60	6,78	9,11	6,92	11,60	26,10	61,50	4
79	w	45	57,3	3	RRYGB	157	0	4	8,2%	-2,7	49,4%	-15,9	14,00	13,20	11,40	6,29	10,59	7,06	8,26	47,00	88,10	5
80	w	26	53,2	2	RRYGB	112	0	3	15,1%	-4,3			13,50	12,80	10,60	9,45	10,77	15,95	4,77	31,50	69,30	3
81	w	52	49,2	3	RRYGB	103	I	3	22,6%	-5,5	41,9%	-10,2	11,00	10,50	11,00	7,55	11,39	9,43	14,80	31,80	72,50	4
82	w	52	55,9	2	RRYGB	126	0	4	14,1%	-4,4			13,20	11,50	12,30	6,04	8,42	6,59	7,85	37,30	55,00	2
83	w	52	40,9	2	RRYGB	75	0	4	22,6%	-3,6			12,20	12,10	11,20	4,92	8,37	5,90	8,25	14,20	16,20	4
84	w	33	48,1	2	RRYGB	87	0	3	15,9%	-3,7	41,9%	-9,7	13,30	13,10	12,90	7,01	9,38	8,18	5,74	18,20	34,50	4
85	w	27	42,2	3	RRYGB	76	0	4	9,4%	-1,6	90,8%	-15,6	12,40	11,90	13,10	6,73	10,20	7,85	4,87	15,40	13,30	4
86	w	24	48,9	2	RRYGB	117	0	4	33,0%	-7,9			13,70	12,40	12,90	8,72	15,85	10,67	2,07	17,40	31,10	5
87	w	42	46,7	1	RRYGB	95	0	4	18,7%	-4,0			14,40	12,40	12,50	9,07	11,13	7,38	3,42	14,70	15,70	4
88	w	48	50,3	2	RRYGB	83	0	4	16,6%	-4,2			11,40	10,20	10,00	4,35	6,70	5,97	1,45	33,40	44,60	4
89	w	35	51,0	3	RRYGB	90	0	3	16,3%	-4,3			13,10	10,60		9,01	11,50		11,10	19,70		5
90	w	35	41,9	2	RRYGB	112	II	4	21,0%	-3,6			12,70	10,70	10,50	5,49	9,23	7,49	11,20	30,20	70,50	6
91	w	31	49,9	2	RRYGB	111	0	3	18,9%	-4,7			15,60	14,30		5,80	7,76		9,52	34,10		11
92	w	26	50,7	1	RRYGB	76	0	3	3,6%	-0,9			13,00	11,60	11,20	5,79	5,65	4,00	4,46	10,80	11,60	3
93	w	49	53,4	3	RRYGB	154	0	3	15,2%	-4,3			15,40	14,50	14,20	5,37	11,06	9,06	5,97	16,40	46,10	6
94	w	43	35,6	3	RRYGB	94	0	4	34,6%	-3,7			14,70	10,80	10,40	7,71	11,78	9,48	19,60	47,10	69,50	4
95	w	22	54,4	2	RRYGB	140	0	3	20,9%	-6,1			15,70	13,60	13,40	10,74	14,47	9,17	7,10	18,00	14,30	2
96	w	46	49,5	2	RRYGB	94	0	4	16,2%	-4,0			13,70	12,30	12,20	5,03	9,43	6,05	2,90	25,20	15,00	3
97	w	39	38,1	4	RRYGB	77	0	4	13,2%	-1,7			12,30	11,50	12,20	8,47	10,81	8,88	13,60	23,90	40,10	4
98	w	62	40,0	4	RRYGB	99	0	3	25,4%	-3,8			15,10	13,60	13,90	10,07	13,10	11,52	7,98	36,20	84,90	5
99	w	44	34,7	3	RRYGB	89	0	3	36,2%	-3,5			14,10	12,40	12,10	11,47	11,40	10,11	5,68	16,40	31,70	5
100	w	44	49,2	4	RRYGB	161	0	4	14,3%	-3,5			13,60	11,80	11,20	8,17	7,19	6,87	4,06	41,20	65,20	
101	w	41	53,9	3	RRYGB	98	0	6	17,2%	-5,0			15,10	14,10	14,00	11,22	14,33	9,28	20,50	36,60	48,10	3
102	w	42	44,8	2	RRYGB	87	0	3	18,5%	-3,7			13,70	12,70		9,69	10,48		8,70	32,30		4
103	w	52	41,8	2	RRYGB	69	0	3	23,2%	-3,9			14,30	11,50	12,50	6,22	9,06	10,96	21,40	47,50	104,00	5
104	w	38	54,9	2	RRYGB	77	0	4	17,3%	-5,2			13,00	10,80	11,90	7,17	7,10	7,18	21,60	28,70	42,80	5
105	w	50	42,6	4	RRYGB	80	0	4	33,5%	-5,9			15,00			7,53			6,02			3
106	w	43	40,7	3	RRYGB	85	0	7	32,7%	-5,1			12,40	10,70	11,00	7,54	6,14	5,38	8,82	41,30	54,40	5
107	w	38	47,8	2	RRYGB	86	0	3	15,2%	-3,5			13,70	12,50	12,30	7,55	9,94	7,64	4,95	26,70	45,20	4
108	w	47	46,7	4	RRYGB	112	0	4	27,1%	-5,9			12,70	11,40	11,30	12,49	8,30	7,57	9,88	38,60	87,30	5
109	w	62	53,5	3	RRYGB	91	0	4	18,5%	-5,3			17,00	15,10	13,80	9,46	11,48	11,01	9,88	46,30	101,00	3
110	w	33	53,3	3	RRYGB	155	0	4	15,6%	-4,4			14,10	11,60	10,50	6,48	7,46	5,59	7,36	35,80	32,80	4
111	w	29	45,4	2	RRYGB	137	0	4	19,6%	-4,0			14,40	12,40	11,90	7,38	11,78	7,27	11,40	15,60	22,30	3
112	w	20	50,3	2	RRYGB	84	0	4	33,6%	-8,5			12,30	10,20	10,10	7,47	10,03	7,04	6,34	15,60	17,00	4
113	w	32	54,0	2	RRYGB	94	0	4	16,5%	-4,8			13,90	14,00	13,60	10,05	15,79	10,28	13,10	38,00	50,00	3
114	w	57	39,6	3	RRYGB	82	0	4	28,8%	-4,2			13,80	11,40	9,12	8,25	8,63	12,00	9,06	15,50	19,50	3
